# Optimizing task‐sharing in abortion care in Ghana: Stakeholder perspectives

**DOI:** 10.1002/ijgo.13000

**Published:** 2020-07-31

**Authors:** Raymond A. Aborigo, Cheryl A. Moyer, Enos Sekwo, Irene Kuwolamo, Eugenia Kumaga, Abraham R. Oduro, John K. Awoonor‐Williams

**Affiliations:** ^1^ Navrongo Health Research Centre Navrongo Ghana; ^2^ University of Michigan Ann Arbor MI USA; ^3^ The Ghana Health Service Accra Ghana

**Keywords:** Abortion, Ghana, Maternal mortality, Medical abortion, Midwife, Surgical abortion, Task‐sharing, Task‐shifting, Unsafe abortion

## Abstract

Ghana has made progress in expanding providers in abortion care but access to the service is still a challenge. We explored stakeholder perspectives on task‐sharing in abortion care and the opportunities that exist to optimize this strategy in Ghana. We purposively sampled 12 representatives of agencies that played a key role in expanding abortion care to include midwives for key informant interviews. All interviews were audio recorded, transcribed verbatim, and then coded for thematic analysis. Stakeholders indicated that Ghana was motivated to practice task‐sharing in abortion care because unsafe abortion was contributing significantly to maternal mortality. They noted that the Ghana Health Service utilized the high maternal mortality in the country at the time, advancements in medicine, and the lack of clarity in the definition of the term “health practitioner” to work with partner nongovernmental organizations to successfully task‐share abortion care to include midwives. Access, however, is still poor and provider stigma continues to contribute significantly to conscientious objection. This calls for further task‐sharing in abortion care to include medical or physician assistants, community health officers, and pharmacists to ensure that more women have access to abortion care.

## Abstract 2

Midwives are providing abortion care in Ghana through task‐sharing; access is still limited, therefore further expansion to include physician assistants and community health nurses is needed.

## 1 Introduction

Inadequate human resources within healthcare systems is common in low‐ and middle‐income countries, especially in hard‐to‐reach areas where maternal mortality is high. The strategic use of midlevel providers through task‐sharing—a process of delegating tasks to less specialized healthcare providers—has been identified as something that increases both productivity and efficiency in health systems.^1^ Task‐sharing can make more efficient use of the human resources currently available by reallocating tasks among healthcare workers; for example, enabling lay and midlevel healthcare professionals such as nurses, midwives, medical doctors, and community health workers to provide clinical tasks and procedures that would otherwise be restricted to higher‐level cadres.^2^

Ghana has expanded nurses’ roles or, in some cases, has provided nurses with additional training to perform tasks that were previously ascribed to only physicians. These are generally midlevel cadres defined by the Ghana Health Service as: “the person trained to support the highly trained health professionals and can hold the fort in the absence of the professional”.^3^ In the early 1990s, midwives were trained in manual vacuum aspiration (MVA) to control uterine bleeding. During that period, a training curriculum for health assistants to support nurses was developed and implemented. In 2002, a strategy to train substitutes for doctors (medical assistants) was proposed and continues to be implemented in selected institutions.^4^ Medical assistants were traditionally professional nurses with only one additional year of training. However, nurses lost interest in the cadre and so a revised training program that takes secondary school graduates was implemented in October 2006.^5^ Physician and medical assistants are still being trained to diagnose and treat various forms of illnesses, from terminal diseases to common colds, to augment the limited number of physicians within the health system.^5^

Accumulated evidence suggests that where abortion is legal on broad socioeconomic grounds and on a woman's request, and where safe services are accessible, unsafe abortion and abortion‐related mortality and morbidity are reduced.^6^, ^7^, ^8^, ^9^, ^10^ The literature further confirms that where there are liberal abortion laws, unsafe abortion and its attendant problems of morbidity and mortality drastically reduce.^10^, ^11^ In Ghana, the abortion law was formulated in 1985. The law allowed abortion under a few conditions, including the impregnation of a “female idiot,” pregnancy as a result of incest or rape, or a pregnancy that threatens the life of the woman or the unborn child.^12^ Since 1985, the law has not witnessed any amendment.

A research study demonstrated the feasibility of using trained midwives at the primary care level to provide postabortion care. It also showed the acceptability of this care by women, healthcare providers, community leaders, and policy makers.^13^ Thus, a reproductive health policy reform by the Ghana Health Service in 2003 allowed midlevel providers with midwifery skills to perform postabortion care in Ghana.^14^ The reform also allowed for abortion care in Ghana to be provided to the full extent of the law; that is, abortion could be provided to protect the physical and mental health and well‐being of a woman on the grounds of rape and where there is fetal malformation.

Over the years, the Ghana Health Service (GHS) has promoted postabortion care within its facilities through the development of guidelines and policies built within the country's reproductive health framework. In 2003, the reproductive health policies and guidelines explicitly included comprehensive abortion care to be provided by trained health professionals with midwifery skills.^14^ This included midwives and medical assistants with midwifery skills. To help ensure that legal abortions are provided safely, the GHS and Ministry of Health developed protocols for the provision of safe abortion. These guidelines, which were adopted in 2006, outlined the components of comprehensive abortion care to include counseling and the provision of contraceptives, defined mental health conditions that could qualify a patient for an abortion, and called for expansion of the provider base by authorizing midwives and nurses with midwifery skills to perform first‐trimester procedures.^15^ To ensure that providers have the necessary skills to offer the service, in 2009 MVA was added to the national curriculum for midwifery education to train additional providers in this lifesaving technique.^16^ The standards were revised in 2012 to reflect the addition.^17^

The 2012 revision of the comprehensive abortion care services task‐sharing policy provided for abortion at various levels of the health system.^17^ These levels included the community, subdistrict, district, regional, and teaching hospitals. It also outlined the providers of services at these various levels to include community health officers (CHOs), nurses, midwives, medical assistants or physician assistants, medical practitioners, and obstetricians/gynecologists who are trained in midwifery and have the necessary skills and ability to perform clinical procedures or tasks that are reproductive health‐related to provide the service.^17^, ^18^ Providers without midwifery skills are limited to referral only. Chemical sellers and pharmacists at all levels are neither permitted to provide abortion services nor manage complications from abortion procedures; however, pharmacists can dispense misoprostol and mifepristone on prescription. Management of complications at the community level by traditional birth attendants and CHOs is limited to referral to the next level of care. At the subdistrict level, midwives and medical assistants with training in midwifery share tasks such as MVA, medical abortion (<9 weeks’ gestation), and management of complications. At the district level, the policy allows task‐sharing among midwives, medical practitioners, and obstetricians. They share tasks such as MVA, medical abortion (<9 weeks’ gestation), and management of complications. Medical doctors and obstetricians at the district level are the only providers permitted to perform dilation and curettage (D&C) and dilation and evacuation (D&E).

Table 11 adapted from the third edition of the “Prevention and Management of Unsafe Abortion: Comprehensive Abortion Care Services Standards and Protocols”^17^ summarizes the legally permissible roles that various healthcare workers have in the provision of abortion care. Required procedures not within their capacity are referred to levels where clients can legally access such services.

**Table 1:** Healthcare workers and abortion services in Ghana

Level	Provider	Task‐sharing					
		MVA	Medical abortion (<9 wk)	Medical abortion (>9 wk)	D&C	D&E	Management of complications
Community	CS	0	0	0	0	0	0
	PH	0	0	0	0	0	0
	TBA	0	0	0	0	0	+
	CHO*	0	0	0	0	0	+
	CHO	+	+	0	0	0	+
Subdistrict	CS	0	0	0	0	0	0
	PH	0	0	0	0	0	0
	MW	+	+	0	0	0	+
	MA*	0	0	0	0	0	+
	MA	+	+	0	0	0	+
District	CS	0	0	0	0	0	0
	PH	0	0	0	0	0	0
	MW	+	+	+	0	0	+
	MP	+	+	+	+	+	+
	OBS	+	+	+	+	+	+
Regional	CS	0	0	0	0	0	0
	PH	0	0	0	0	0	0
	MW	+	+	+	0	0	+
	MP	+	+	+	+	+	+
	OBS	+	+	+	+	+	+
Teaching hospital	MW	+	+	+	0	0	+
	MP	+	+	+	+	+	+
	OBS	+	+	+	+	+	+

Abbreviations: CS, chemical seller; PH, pharmacist; TBA, traditional birth attendant; CHO*, community health officer (not trained in midwifery); CHO, community health officer; MW, midwife; MA*, medical assistant (not trained in midwifery); MA, medical assistant; MP, medical practitioner; OBS, obstetrician; MVA, manual vacuum aspiration; D&C, dilation and curettage; D&E, dilation and evacuation; +, activity is done; 0, activity not done.

Source: Reproduced with permission obtained from the Ghana Health Service.^17^

Over the years, the abortion policy has witnessed many reviews and discussions with the goal of making abortion care more accessible, affordable, and of better quality for the average Ghanaian woman. Although Ghana has made significant strides in expanding the cadres of providers for abortion care, access to the service is still a challenge as midwives are not present in most primary care facilities.^19^ A recent assessment of Ghana's Community‐based Health Planning and Services (CHPS) program showed that nationwide, about 15% of CHPS compounds have midwives compared with 42.4% with a CHO.^19^ This implies that further task‐sharing to include CHOs could improve access by about 27%.

The aim of the present study was to explore stakeholder perspectives on the process of task‐sharing in abortion care in Ghana—including facilitating factors and barriers—and the opportunities that exist to optimize the strategy to improve access to abortion services.

## 2 Materials and Methods

### 2.1 Study setting

The study was implemented in Ghana, which has a population of about 25 million.^20^ About 20% of women of reproductive age (15–49 years) have ever had an abortion.^21^ In 2017, 53 114 abortions occurred and, of these, 13 918 were characterized as unsafe. Nonmedical methods (e.g. drinking milk/coffee/alcohol/other liquid with sugar, drinking a herbal concoction, drinking other home remedies, using a herbal enema, inserting a substance into the vagina, heavy massage, excessive physical activity, and use of all kinds of unknown tablets) used to induce abortion make up more than 27% of abortions carried out.^21^ More than one in 10 pregnancy‐related deaths occur as a result of an unsafe abortion and for every woman who dies from an unsafe abortion it is estimated that 15 suffer short‐ and long‐term morbidities.^21^

Countrywide estimates may mask regional differences; for example, 14% of pregnancies among women in urban areas end up in an induced abortion compared with 7% among women in rural areas. Women in poor and rural communities in northern Ghana have less access to comprehensive abortion care; for instance, only 3% of women in the northern part of the country have ever had an induced abortion compared with 22% of women in the more urban middle and coastal areas.^21^

### 2.2 Study design

This was an exploratory, descriptive study designed to gain insights into the policy decision to include all cadres of health workers with midwifery skills in the provision of abortion care, and to learn about stakeholder opinions regarding opportunities for further expansion.

### 2.3 Sampling of respondents

We purposively sampled individuals in the public and private sector who had themselves contributed (or whose agencies had contributed) to the policy on the expansion of abortion care to include midwives. Selection was based on an individual's or agency's role in advocating for the policy or contributing to the policy framework, implementation, monitoring, or evaluation. Respondents were typically heads of the agencies but, where necessary, members who were more familiar with the agency's role in task‐sharing in abortion care were invited for the interview. Identification of the agencies was primarily via snowball sampling.

### 2.4 Data collection

The lead researcher (RAA) conducted most of the interviews. A research assistant (EK) with more than 2 years’ experience in conducting qualitative interviews assisted him. In all, 12 key informant interviews were conducted. On two occasions, the study team had to talk to two individuals from an agency to better understand the agency's contribution to the policy shift. Consent was sought from the respondents to audio record the interviews. The interviews lasted 1–1.5 hours. All interviews were conducted in June 2018.

### 2.5 Data processing and analysis

All interviews were audio recorded and transcribed verbatim. The transcripts were imported into NVivo version 11 software (QSR International; Melbourne, Vic., Australia) for coding and thematic analysis. We predetermined codes using the interview guide and additional codes were developed for concepts that were not initially captured by the guide but emerged inductively from the data. We segmented the data into similar groups to form preliminary categories of information or themes on the expansion of health worker roles in abortion care. We examined the segments of data related to each theme and where necessary refinements were made.

RAA coded all transcripts and ES coded six of those transcripts separately. The two coding sets were compared to ensure validity. Discrepancies were discussed and coding was adjusted where necessary. A coding comparison query to determine the inter‐rater reliability returned a Kappa coefficient of 0.84.

## 3 Results

Stakeholders were asked to comment on five main thematic areas: (1) the motivation underlying task‐sharing for abortion services; (2) their own roles in advocating for task‐sharing; (3) factors that facilitated task‐sharing in abortion care in Ghana; (4) barriers to task‐sharing in abortion care; and (5) opportunities to task‐share beyond the midwife.

### 3.1 Motivation to task‐share in abortion care

Stakeholders described the health system's increasing understanding that not every pregnancy is desired and that women in desperation are likely to attempt all manner of procedures to get rid of unwanted pregnancies. In that regard, they commented that the health system had a duty to provide for the needs of all groups of people by increasing both geographical and financial access to the service. For example, it was realized that some district public hospitals have only one doctor working with a number of midwives, and when the doctor goes on leave, people in need of abortion services are denied access when the midwives could easily provide it.

Stakeholders also described how the government was increasingly concerned about morbidity and mortality resulting from induced abortion and pregnancy‐related complications. One respondent reported high maternal deaths related to abortion complications at the Tamale Teaching Hospital in the northern region of Ghana. He observed that instead of accessing abortion care from health facilities, women were doing so from untrained persons. Consequently, in the 1990s, the health service revised the reproductive health policy to include the provision of safe abortion services to improve access for those who need it. A stakeholder from the GHS revealed:

Abortion probably at some stage was the second leading cause of death among pregnant women. You always see hemorrhage and then a lot of the time abortion comes even before pre‐eclampsia so it was a concern and it was something that was preventable either with family planning or safe abortion services. Untrained people were offering unsafe abortion because we [GHS] didn't offer the service. They went to quacks and barbers and all sorts of characters who were doing it for them. Taking herbal concoctions, dangerous poisons just to induce abortion and so by offering the service we averted all those deaths. Key informant interview, GHS01

### 3.2 The role of the stakeholders

Respondents indicated that the push for task‐sharing in abortion care was the initiative of the GHS. The Ministry of Health is responsible for policy decisions while the GHS is the agency responsible for implementation, with the support of a number of partner NGOs. The excerpt below summarizes the role of the GHS:

…That is our job and everybody is just helping us… If you look at our policy and standards, by 2002/2003 when we did our second review, it said provide abortion care services—safe abortion care services, as permitted by law. …[W]e are in the lead; they [partner NGOs] are just here to help us implement our policy. Key informant interview, GHS02

Ipas Ghana approached the Nursing and Midwifery Council and suggested that midwives could safely provide comprehensive abortion care in Ghana based on evidence from other countries. Ipas then worked with the GHS to develop the standards and protocols and assisted in reviewing the curriculum for nursing and midwifery training institutions to include comprehensive abortion care. Ipas reported that although they succeeded in pushing for preservice training in comprehensive abortion care, the didactic training was not sufficient in practice; thus, those interested in providing comprehensive abortion care receive an additional 2 weeks of hands‐on GHS‐certified training before they are allowed to practice.

Other agencies also played key roles in bringing about the inclusion of midwives in abortion care, as described by a key informant from the Population Council:

We've been at the forefront of trying to shape policy… we've been working very closely with the Ghana Health Service. We started with five international NGOs. Right now they are four and right now two of the organizations are working in the area of increasing access to safe abortion. One working in the public sector, Ipas, and one in the private sector, Marie Stopes, and we coordinate that group and work very closely with the Ghana Health Service. Out of this consortium that is where the task‐sharing with the midwives to be able to provide the abortion services came from. Key informant interview, Population Council

While Ipas supports the GHS to train midwives in the public sector, Marie Stopes International (MSI) and the Planned Parenthood Association of Ghana (PPAG) support training in the private sector. PPAG and MSI have clinics across the country that provide family planning and abortion services using mostly midwives.

Ipas is currently advocating for free abortion care for women or girls who qualify under the law to have the service. These include women and girls who have been raped, those whose lives are threatened by the development of the fetus, and those whose mental faculties are affected by the pregnancy. Ipas is of the view that the service should be covered under the National Health Insurance Scheme (NHIS). They argued that no woman has sex just to have an abortion; thus, if women are faced with the difficult decision to have an abortion, they should have it for free and they should receive adequate counseling to avoid a repeat abortion due to an unwanted, unplanned, and mistimed pregnancy. Abortion care should be treated as any other medical procedure, as described by an Ipas interviewee:

When you have a headache, you go to hospital, when you have malaria you go to hospital, when you need a termination you should also go to the hospital, take a folder and go through the process like any other medical condition. Key informant interview, Ipas

### 3.3 Facilitating factors for task‐sharing in abortion care in Ghana

Stakeholders recounted that, in the past, abortion was performed by doctors because it was done in the theater using curettes. However, when therapies such as MVA and medications became available, leaders in the health system realized that they could use the absence of a clear definition of “medical practitioner” under the law to include midwives to provide abortion care. According to the stakeholders, Ghana's law on abortion care only permitted medical practitioners to provide the service, which vaguely suggested “medical officers” were the only ones allowed. According to stakeholders, the ratio of medical officers to the population who needed the service was highly disproportionate. In addition, there were reports that medical doctors are not typically frontline providers and they are not accessible, especially in rural areas. This therefore presented an opportunity to expand the providers to include midwives within the community to open the frontiers for more people to have access to safe abortion services.

I think they took advantage of the law being flexible and then started pushing, advocating for the provision of the service. You know formerly it was only gynecologists who were providing abortion services; they wouldn't even allow the midwives. But looking at the laws vis a vis the standards and protocols and the guidelines of the Ghana Health Service that is why other cadres of health service providers, like the midwives, were brought in. Looking at the first trimester of abortion, it is not very complicated when it comes to providing abortion services. So they took advantage of that and added the midwives and trained them to provide the service. Of course before then a lot of advocacy had been done. Key informant interview, PPAG

For most stakeholders, midwives are already using skills that they acquired during their training to save the lives of women with complications and therefore including them in abortion care was not new.

For comprehensive abortion care, they are midwives they have the skills already; what you needed to do to them was to give them additional training; they are already in their domain of training. If they are to do deliveries they enter the uterus anyway, they do hand manual removal of the placenta so they know the anatomy of the female organ, they know what the uterus is, they know what the tubes are. When a woman ruptures they know, so that is their domain; the addition is the additional procedure. Key informant interview, GHS01

Stakeholders also felt that there is a high number of midwives and Ghana's flagship program for primary health care^22^ allows for the placement of midwives in communities, thus making them more accessible to rural residents than medical doctors.

Furthermore, there was no overt opposition to the inclusion of midwives in abortion care. Stakeholders said that although the initiative saw some doctors who were motivated by their religious beliefs (e.g. Jehovah Witnesses) threatening to take the law to court to seek an interpretation of the term “medical practitioner,” no group officially opposed the expansion of abortion care to include midwives. The Ghana Medical Association did not oppose the policy, as was articulated by one of the respondents:

No, there has not been any official resistance. As I indicated, there were a few individuals who opposed the midwives in their facilities from providing the services but it is not on a large scale and the Ghana Medical Association has not come out to condemn the inclusion of midwives. There has not been any official statement or authority from the Ghana Medical Association; they rather want the service to reach every corner of the country. Key informant interview, Komfo Anokye Teaching Hospital

Stakeholders also identified a group of influential Ghanaians who were brought together as champions for the task‐sharing policy. They said members of the group were at the frontline, on TV and radio programs, managing the backlash that stemmed from powerful individuals and groups. In addition to explaining the policy to health facility staff, respondents indicated that there were regular mass education campaigns on radio, TV, and other public platforms to sensitize the population to the policy and to inform them about the availability of the service. Stakeholders who were engaged during the process included lawmakers, judges, the police, traditional leaders, women's groups, and religious bodies. The media was also cautiously included as a powerful tool for advocacy.

Interviewees reported that financial access to abortion care was limited because medical doctors exploited clients in need of abortion services by charging huge sums of money before providing it. The health authorities therefore used the inclusion of midwives to standardize charges for abortion services. Clients now pay 14–15 Ghana Cedis (USD $3) for an abortion instead of paying fees at the discretion of doctors, which ranged from 200 to 2000 Ghana Cedis (USD $40–400) as reported by this stakeholder:

And there is no regulation as to how many months, oh its two months oh he says ok then pay 1000, four months old I will charge you 2000. It is a sort of bargaining procedure and they are getting a lot of money and anything above one month 150 Ghana Cedis or 200 Ghana Cedis or 1000 Ghana Cedis so people are making money. Key informant interview, GHS01

### 3.4 Barriers to task‐sharing in abortion care in Ghana

According to stakeholders, owing to the stigma around abortion, some women still prefer “quacks*”* because the health facility environment does not ensure privacy and confidentiality. “Quacks*”* were defined as all providers both formal and informal who are not trained to provide safe abortion care.

Furthermore, respondents identified conscientious objection as one of the barriers to abortion care. They acknowledged the difficulty in eliminating it from the service because there will always be people with strong opposing views. There were reports that some midwives refuse to provide the service after attending the trainings because relatives, especially their spouses, did not approve of it. Others stopped providing the service because their pastors preached against it. In some communities, providers are stigmatized by both community members and their colleagues as “abortion nurses” and all their properties are tagged as things bought with “abortion money.” In addition, community members believe that female abortion providers who have challenges with childbirth are cursed because they provide abortion services. Some of these providers are conscientious objectors because they are undergoing medical procedures to get pregnant while part of their job is to abort pregnancies.

What is really a problem with abortion in Ghana is stigma; the judgment. Even a married woman to go and have abortion has some reservation in terms of stigma and that stigma is not self‐stigma; most of the time, it's the client service provider stigma. The stigma, once you are pregnant and you want to have an abortion it means you have been promiscuous. If I'm a married woman, I have a child eight months and I realize that I am pregnant again and go for abortion there is still some stigma, judgmental. Why did you allow yourself to get pregnant after eight months? It means that you are promiscuous or you like sex too much. …you know your psyche; abortion means that you had sex and sex is not a good thing to talk about ‐ that is the challenge. Key informant interview, GHS01

Stakeholders described how, as part of the policy, providers are paired with mentors who hold mentor–mentee meetings to discuss challenges, including conscientious objection. They reported that, through the values clarification exercise, agencies assist providers to transform their attitudes toward the practice through a better understanding of the dynamics of abortion to make them more comfortable to provide the service.

### 3.5 Beyond the midwife

Some stakeholders reported that, in the absence of complications, other levels of medical practitioners should be allowed to provide the service because the current expansion is not adequate. According to some, demand for abortion services still outstrips supply, especially in rural areas; meanwhile, opportunities still exist for task‐sharing in abortion care within the healthcare system. For instance, most of the stakeholders were of the view that even though medical or physician assistants do not have midwifery training, they have been conducting deliveries; thus, they could be trained to provide abortion care.

It's possible to go a step further especially if you look at medical abortion. As long as we can build the needed capacity for people to be able to determine the age of pregnancies adequately and other related things like picking up ectopic pregnancies and all that, then yes we might get to a point where we want to go beyond midwives just for medical abortion. Because surgical abortion we might need much longer time to do that. So medical assistants are clearly one cadre that we can use, so you go to a typical public facility, the medical assistant supervises the midwives. At a typical health center for instance where there is a medical assistant and there is a midwife, so the medical assistant is considered head of the facility and the midwife works under the medical assistant. And yet when it comes to the law, the medical assistant is not allowed to provide abortion care and the midwife is allowed. Key informant interview, MSI

Stakeholders reported that the suggestion to include medical assistants in abortion care is based on anecdotal evidence that male medical assistants were providing abortion services that were unsafe and, therefore, streamlining their activities and equipping them with the necessary skills could further save lives. Agencies like Ipas are advocating for the government to include medical assistants as part of the task‐sharing policy in abortion care, but the push has seen very little success. A respondent from the GHS felt that inclusion of midwives was sufficient and there was no need to include other cadres of health workers.

If you look at the policy I showed you, the policy is that all midwives are allowed to do so there are many midwives that we can continue to train. Many midwives come out all the time so we will continue reviewing that. We don't need to take a new cadre but there are enough midwives who are everywhere. Those who are willing and ready, we train them to offer the service. Key informant interview, GHS02

The health authorities were divided on this issue, as another respondent from the service said:

They are getting pregnant in the villages, in the communities, so again I don't see why a CHO who has gone for two years training cannot be trained two weeks in addition [to provide the service]. Key informant interview, GHS01

This view was supported by other stakeholders who argued that community health nurses who are more accessible in rural communities can be trained to provide medical abortion to further improve access to abortion care.

Other cadres of health workers, such as professional pharmacists, are also being considered by some agencies to provide medical abortion because they believe that trained pharmacists who can give medical advice should be allowed to prescribe, sell, monitor and, if they see complications, refer to hospitals.

## 4 Discussion

The global initiative on task‐sharing has helped countries make more efficient use of their human resources for health by reallocating tasks among healthcare workers to allow lay and midlevel healthcare professionals to provide clinical tasks and procedures safely that would otherwise be restricted to higher‐level cadres. Ghana has been successful in sharing clinical responsibilities between medical doctors and midwives partly because the global initiative on task‐sharing supported it. The policy to expand health workers in abortion care to include midwives was also within the remit of the law. The GHS and partners leveraged the liberal law on abortion and the lack of clarity on terminologies such as “medical practitioner” to include midwives in abortion care.

Several studies on task‐sharing have made recommendations that healthcare providers should be trained in services close to their job descriptions to make task‐sharing more efficient.^23^ In Ghana, midwives had comprehensive training that supported the organization of task‐sharing in abortion care. The additional 2 weeks’ training certified by the GHS was to hone the skills of the midwives. Provision of additional training for midlevel providers in abortion care before they provide the service has been recommended in other settings.^24^ Thus, their inclusion in abortion care did not raise concerns about lowering standards of care or lowering the distinction of doctors who have dedicated many years to earn their professions. Studies in sub‐Saharan Africa have shown safe outcomes for midlevel providers such as nurses, physician assistants, and midwives trained in medical abortion services.^25^, ^26^

Opportunities still exist within the GHS for further expansion of health worker roles in abortion care. Current advocacy efforts focus on the inclusion of medical or physician assistants, pharmacists, and community health nurses in abortion care to improve access and further reduce the contribution of unsafe abortion to maternal morbidity and mortality. With the evolution of medical therapies, women do not necessarily need a medical doctor to have a safe abortion, although good dating of a pregnancy should remain a priority. Indeed, some studies have suggested that women can self‐administer medical abortion medicines safely and effectively via telemedicine.^26^ In view of that, midlevel providers such as nurse practitioners and physician assistants should be included in the health workforce that provides abortion care. If trained, these midlevel providers can provide first‐trimester MVA and medical abortion as safely and effectively as physicians and midwives.^26^, ^27^ Because CHOs work in remote communities, it might not always be practical to refer women in need of abortion care to other facilities—most may have difficulty traveling the long distances or meeting the costs of travel. Under these circumstances, CHOs may be forced to provide the service outside of their defined tasks owing to the absence of a midwife or doctor. A study in central Uganda reported similar findings for midwives before their role in abortion care was formalized.^24^ Furthermore, even though the abortion policy does not permit pharmacists to provide abortion, they still do. According to the 2017 maternal health survey, doctors, nurses, or community health officers/nurses are the most common abortion providers (41%) followed by pharmacists, who provide 33% of abortions.^21^ Further task‐sharing to include CHOs and physician assistants, as well as pharmacists for medical abortion, is therefore feasible and should be pursued by the health system.

In conclusion, task‐sharing in abortion care has been embraced by the health service in Ghana to improve access to safe abortion services. Factors such as availability of data on the contribution of unsafe abortions to maternal deaths contributed to the rapid inclusion of midwives in abortion care. Provider stigma still contributes to conscientious objection but strategies such as values clarification are helping to get more health workers to provide the service. Considering that midwives and doctors are in limited supply, coupled with the high prevalence of conscientious objection,^28^ not all midwives and doctors will offer the service. Therefore, there is a need to continue to expand health worker roles in abortion care to include providers such as medical or physician assistants, CHOs, as well as pharmacists, to ensure that more women—especially those in rural areas—have access to safe abortion care.

## Author Contributions

RAA designed the research, planned the data collection, conducted the research and data analysis, and led the manuscript writing. CAM contributed to the analysis and manuscript writing. ES, IK, and EK contributed to planning, data collection, and analysis. ARO critically reviewed the paper and made significant inputs to data analysis and interpretation. JA contributed to planning, conduct, data analysis, and manuscript writing. All authors read and approved the final version of the manuscript.

## Conflicts of Interest

The authors have no conflicts of interest.
